# EHMTI-0355. Comparison of carbamazepine and oxcarbazepine tolerability in patients with trigeminal neuralgia

**DOI:** 10.1186/1129-2377-15-S1-I2

**Published:** 2014-09-18

**Authors:** R Cregg, E Besi, D Boniface, J Zakrzewska

**Affiliations:** 1 Pain medicine, University College London, London, UK; 2 Facial Pain Unit, Eastman Dental Institute, London, UK; 3 Health Behaviour Research Centre, Eastman Dental Institute, London, UK

## Introduction

Little work has been done around the adverse events profile (AEP) of drugs used to treat trigeminal neuralgia (TN). TN patients are often unaware of significant side effects associated with pharmacotherapy.

## Aims

To examine the AEP of carbamazepine and oxcarbazepine in TN patients.

## Methods

74 TN patients averaging 1.76 outpatient consultations over 2 years and undergoing treatment only with carbamazepine or oxcarbazepine were recruited to complete the AEP questionnaire (Baker et al., 1994) at each outpatient visit in relation to the current drugs being taken for pain control. The AEP contains 19 items assessing the frequency of a range of adverse effects using a scale of 1 to 4, with 4 indicating more frequent occurrences. Scores can range from 19 to 76 and >45 suggests toxicity. Drug dosages were converted to mg/kg. Efficacy of 200mg dose of carbamazepine is considered equivalent to 300mg oxcarbazepine (Beydoun 2002).

## Results

Using a multilevel logistic regression model, 50% probability of significant toxicity for a 70kg person is estimated at 1300 mg carbamazepine and 2600mg oxcarbazepine. Women reported higher levels of toxicity. Most common side effects were tiredness, memory problems, sleepiness, difficulty in concentration, unsteadiness. Hyponatraemia occurred more frequently in patients on oxcarbazepine and is dose related.

## Conclusions

At equivalent clinical doses oxcarbazepine results in fewer AEPs than carbamazepine and so should be considered as an alternative to carbamazepine in case of intolerance.

No conflict of interest.

